# The Brain and Early Experience Study: Protocol for a Prospective Observational Study

**DOI:** 10.2196/34854

**Published:** 2022-06-29

**Authors:** William Roger Mills-Koonce, Michael T Willoughby, Sarah J Short, Cathi B Propper

**Affiliations:** 1 School of Education The University of North Carolina at Chapel Hill Chapel Hill, NC United States; 2 Research Triangle Institute Research Triangle Park, NC United States; 3 School of Education University of Wisconsin at Madison Madison, WI United States; 4 Frank Porter Graham Child Development Institute The University of North Carolina at Chapel Hill Chapel Hill, NC United States

**Keywords:** poverty, executive functioning, parenting, language, sleep, neurological development

## Abstract

**Background:**

Children raised in conditions of poverty (or near poverty) are at risk for nonoptimal mental health, educational, and occupational outcomes, many of which may be precipitated by individual differences in executive function (EF) skills that first emerge in early childhood.

**Objective:**

The Brain and Early Experience study considers prenatal and postnatal experiences that may mediate the association between poverty and EF skills, including neural substrates. This paper described the study rationale and aims; research design issues, including sample size determination, the recruitment strategy, and participant characteristics; and a summary of developmental assessment points, procedures, and measures used to test the study hypotheses.

**Methods:**

This is a prospective longitudinal study examining multiple pathways by which poverty influences normative variations in EF skills in early childhood. It is funded by the National Institute of Child Health and Human Development and approved by the institutional review board.

**Results:**

Recruitment is complete with a sample of 203 participants, and data collection is expected to continue from September 2018 to February 2024. Of those recruited as *low socioeconomic status* (SES), 71% (55/78) reported income-to-needs (ITN) ratios of <2.0, and 35% (27/78) reported ITN ratios of <1.0. Among participants recruited into the *not-low SES* stratum, only 8.8% (11/125) reported ITN ratios of <2.0, and no participant reported ITN ratios of <1.0. The average ITN ratio for participants recruited into the low-income stratum was significantly lower than the average for the high-income recruitment cell (*P*<.001). Comparable recruitment outcomes were observed for both Black and non-Black families. Overall, the sample has adequate diversity for testing proposed hypotheses, with 13.3% (27/203) of participants reporting ITN ratios of <1 and >32.5% (66/203) reporting ratios of <2.0.

**Conclusions:**

Preliminary results indicate that the recruitment strategy for maximizing variation in family SES was successful, including variation within race. The findings of this study will help elucidate the complex interplay between prenatal and postnatal risk factors affecting critical neurocognitive developmental outcomes in early childhood.

**International Registered Report Identifier (IRRID):**

DERR1-10.2196/34854

## Introduction

### Background

On average, children raised in poverty perform more poorly on cognitive assessments and achieve lower academic outcomes than children from higher-income families [1], possibly owing to the effects of nonoptimal environmental experiences compromising early brain maturation and executive function (EF) skills [2]. Despite its significance, limited information exists on the neural and cognitive precursors and the social determinants of EF skills, especially during the first 3 years of life [3]. To address this scientific gap, the Brain and Early Experience (BEE) study examines specific prenatal and postnatal pathways from economic disparity to EF skills mediated by environmental experience and early structural and functional brain development.

### Study Design Considerations

Beginning in utero, poverty imposes numerous risk factors [4] that contribute to an achievement gap persistent throughout formal schooling for poor and nonpoor children [5]. This leads to less educational attainment, increased likelihood of single parenthood, lower occupational status, poorer physical and mental health, and increased risk for all causes of mortality [6]. The limited development of neural substrates supporting neurocognitive development and the disrupted or delayed emergence of EF skills and other cognitive and language abilities may serve as mechanisms by which poverty gets *under the skin* and alters developmental trajectories across the life span [7].

A variety of cognitive processes that support goal-directed behavior are subsumed under the construct of EF. In early childhood, the 3 most widely studied subdomains of EF include *working memory*, defined as the holding in mind and updating of information while performing some operation on it; *inhibitory control*, defined as the inhibition of prepotent or automatized responding when engaged in task completion; and *mental flexibility*, defined as the ability to shift attentional or cognitive set among distinct but related dimensions or aspects of a given task [8]. Identifying the specific experiential and neurocognitive mechanisms through which poverty leads to poor EF is essential for optimizing early intervention programs.

Neuroscientists investigating how poverty influences children’s neural development have identified systematic differences in structural brain development that mediate associations between poverty and impaired academic outcomes [9-12]. Specific examples include developmental differences in the maturation of frontal and temporal lobe gray matter that explained up to 20% of the variance in low-income children’s cognitive deficits [13] and different surface-based morphometry indexes (ie, cortical thickness, surface area, cortical folding, and combinations of these) between poor and not-poor children [3]. These brain regions (and associated neural networks) may be highly vulnerable to the early environmental risks associated with poverty. Therefore, the BEE study applies a developmental science approach to the study of prenatal [14] and postnatal [15] determinants of neurocognitive development and later EF.

As the earliest experience in development, the prenatal period is a highly sensitive time for neurocognitive development. Although there are numerous studies on women’s physical health during pregnancy and offspring neurodevelopment [16-18], there is less research on how mental health and psychological experiences during pregnancy may be associated with subsequent neurodevelopmental outcomes. Factors such as elevated prenatal stress may influence early neurocognitive skills through their influence on the proliferation, differentiation, migration, and aggregation of fetal neurons [19,20]. For example, Buss et al [21] report that pregnancy-specific anxiety predicts reduced gray matter density in the cortex, the left middle temporal lobe, the entorhinal cortex, and the parahippocampal gyrus at 6 to 9 years of age. The BEE study examines both subjective measures of prenatal stress (via self-report) and objective measures (via biological stress markers) as potential mediators of associations between poverty and prenatal brain development.

Following birth, poverty adversely influences children’s development through early proximal experiences [22], with negative associations reported between family socioeconomic status (SES) and caregiving behaviors [23,24], child sleep quality [25], and child language exposure [26,27]. In contrast, multiple studies indicate that the quality of early caregiver–child interactions predict childhood EF and changes in EF throughout time [28-30]. These effects may be owing to the support and stimulation provided by a responsive parent or the favorable environment in which children can practice these developing skills as active agents in their own learning and skill acquisition. Similarly, sleep hygiene in early childhood predicts emerging EF in children [31]. Bernier et al [32] report that sleep in the first year of life predicts improved EF at 26 months; however, it was not related to other cognitive outcomes such as verbal ability or broader cognitive functioning, suggesting that sleep may be particularly important for EF. Activities that engage executive control are effortful and may be supported through energy restoration that occurs during sleep [33,34]. Finally, early language exposure contributes to children’s emerging language and EF skills [35,36]. Children’s expressive language contributes to problem solving and self-directed speech to regulate thoughts, emotions, and behavior [37,38] and emerging EF skills [39-44].

Considering these findings, the BEE study examines caregiver–child interaction quality, child sleep hygiene, and language exposure as environmental experiences potentially mediating the associations between poverty, brain development, and emerging EF. These constructs were also selected because they (1) are among the most salient for all children and account for a large percentage of the child’s daily lived experiences, (2) have reliable, valid, and developmentally appropriate methods of measurement across time, and (3) are moderately stable throughout time, providing confidence that our assessments will represent the overarching experience of the first 3 years of life. It is also important to note that extreme levels of poverty are not necessary for observing the associations between family income and child outcomes. Several studies report that children living above but near the poverty level—or even up to 2 times the poverty level—experience significant neurocognitive risk [9,12]. As described further, the BEE study purposefully sampled families along a range of socioeconomic risks (with oversampling for those in poverty) to capture the variability in poverty and poverty-related risk predictive of neurological and EF development.

The BEE study also uses state-of-the-art functional magnetic resonance imaging (fMRI) measurements to observe neurological development soon after birth and before the third year of life. Most studies examining associations between poverty and the brain used global metrics of brain development (eg, total gray matter and total surface area) and relied on cross-sectional data and mixed age samples. However, the development of EF skills involves efficient information processing between brain regions relying on the integrity of specific white matter tracts and the underlying neural networks connecting them [45]. Moreover, it may be that the developmental pattern during this earliest period of life is most informative, given that the most prolific changes in brain development that inform early EF occur from birth to 3 years [46]. The BEE study addresses these limitations by examining associations between poverty and early structural (ie, white matter integrity via diffusion tensor imaging [DTI]) and functional (ie, resting state [rs] networks via fMRI) aspects of brain development at 2 weeks and 30 months of age. In this study, we will interpret brain development at 2 weeks of age as a product of prenatal experiences and brain development at 30 months of age as a combination of prenatal and postnatal experiences.

### Aims and Hypotheses of the BEE Study

**Figure 1:**
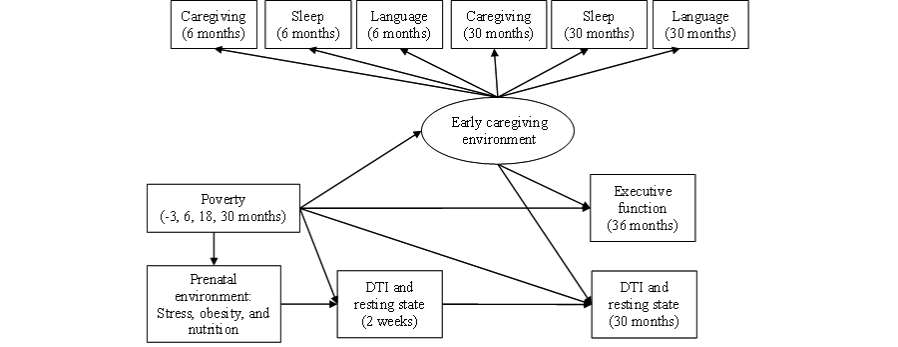
Conceptual model depicting research aims. DTI: diffusion tensor imaging.

The BEE study addresses 3 specific aims. Aim 1 involves the examination of associations between poverty and neonatal structural and functional brain development at 2 weeks of age. Two sets of hypotheses are proposed for aim 1. The first is that poverty will predict individual differences in neonatal structural brain development (specifically for white matter tracts that support cognitive processes of emerging EF) and functional brain development (including rs networks related to attention, salience, executive control, and default mode); the second is that these associations will be partially mediated by prenatal experiences, including stress, nutrition, obesity, and toxic environmental exposures. Aim 2 involves the examination of associations between poverty and toddler structural and functional brain development at 30 months of age and potential mediators of these associations. Three sets of hypotheses are proposed for aim 2. The first is that poverty will predict individual differences in changes in toddler structural and functional brain development. The second hypothesis is that prenatal experiences will partially mediate these associations. The third hypothesis is that postnatal experiences (ie, caregiving, sleep hygiene, and language exposure) will partially mediate the association between poverty and toddler brain development. Finally, aim 3 involves the examination of pathways from poverty to EF at 36 months of age. Three sets of hypotheses are proposed for aim 3. The first hypothesis is that poverty will be negatively associated with EF, the second is that prenatal experiences will partially mediate the association between poverty and EF, and the third is that postnatal experiences will also partially mediate these associations. The path model in Figure 1 illustrates these aims and hypotheses.

## Methods

### Sample

#### Power Analyses to Determine Sample Size

Two Monte Carlo studies were conducted to determine the statistical power to evaluate study aims given the proposed sample size and design. Both studies, which adopted the approach of Thoemmes et al [47], included a population generating model of 200 participants, assumed a type I error rate of 0.05, and involved 5000 replications. With these parameters, for tests of main effects (encompassing associations between poverty child outcomes, including brain development at 2 weeks and 30 months and child EF, general cognitive ability, and language development at 30-36 months), the sample has the power (≥0.98) to detect small to medium (*R*^2^=0.075) sized main effects but has limited power (0.51) to detect small (*R*^2^=0.02) main effects. For tests of mediation (encompassing the main effects as mediated by prenatal and postnatal experiences), the sample has power (>0.99) to detect single mediated effects when both the *a* and *b* paths are of medium size (*R*^2^=0.13). The study has power (>0.89) to detect a mediated effect when both *a* and *b* paths are of small to medium size (*R*^2^=0.075). However, the power to detect a mediated effect drops to 0.45 when either *a* or *b* paths are of small size (*R*^2^=0.02), even if the other path is of medium size. As a point of reference for those readers who are unfamiliar with Monte Carlo methods, 200 results in a power of 0.80 to detect bivariate correlations |*r*| ≥0.20, which also corresponds to a small to medium–sized effect per the Cohen conventions [48]. The study will rely on high-quality measurement and repeated measures to improve the magnitude of the effects.

#### Recruitment Strategy

From August 1, 2018, through October 31, 2020 (26 months), we used multiple methods to reach the population of interest (pregnant women in their second trimester). These include targeted advertisements on social media, online community message boards, hospital records in the local university-based medical center, listserv emails to local university staff and students, paper fliers posted in obstetrician–gynecologist offices in the local geographic area, and personally staffing information tables at the Women, Infant, and Children centers before, during, and after local pregnancy and birthing classes. Individuals interested in participating in the study were directed to a brief web-based survey that asked questions targeting inclusion criteria, including being currently pregnant with a single fetus and speaking primarily English at home. If both criteria were met, subsequent questions regarding due date, contact information, and permission to be contacted for participation were asked. Next, a study personnel used this information to schedule a phone call with each participant. During this phone call, the researcher confirmed the inclusion and exclusion criteria and obtained the person’s address to confirm that they lived within a 45-minute radius of the study location and that there was no intent to move out of the geographic area in the next 3 years.

The research staff then asked demographic questions to assign potential participants to 1 of 4 recruitment cells based on a 2×2 (SES×race) design. This categorization as part of the recruitment process was important because, as a study focusing on the role of poverty in early development, we sought to limit confounding between income and race by oversampling (relative to local family demography) for combinations of (1) lower SES and non-Black racial identity and (2) not-low SES and Black racial identity. Three demographic questions were asked for all potential participants who met inclusion criteria. The first question was about the person’s race and ethnicity. The second question was about the individual’s highest level of education. The third question asked about the use of a range of federal social services that are eligible to families at or below 185% of the federal poverty level, including Women, Infant, and Children program, Temporary Assistance for Needy Families, Supplemental Nutrition Assistance Program, or Medicaid. Following the precedent established by other large-scale developmental studies of families living in poverty [49], we used these questions to assign potential participants into a *low-SES* group if they had no education beyond a high-school diploma or general education degree or if they currently used a social service. Otherwise, they were classified as *not-low SES*. We designated a participant as *Black* if they self-identified as Black, African American, or multiracial Black; otherwise, they were designated as non-Black. These 2 dimensions were cross-tabulated to create 4 target recruitment cells with the goal of recruiting comparable numbers of low-SES and high-SES families within each racial group to reduce potential confounding between race and family income. The assignment of families into cells was intended solely to guide recruitment efforts and should not be used for hypothesis testing regarding family income and race. Families were considered formally enrolled if they completed 2 of the first 3 data collection visits (including the prenatal visit, the 2-week postnatal visit, and the 6-month postnatal visit). If a family did not meet these inclusion criteria, they were dropped from the study and replaced by a newly recruited family with comparable SES and racial identities. See Multimedia Appendix 1 for a flowchart documenting recruiting and sampling numbers that resulted in the final sample size of 203 participants.

#### Sample

On average, biological mothers of BEE participants were aged 30.53 (SD 5.35, range 18-46) years and had completed 15.36 (SD 2.66) years of education at the prenatal visit. Approximately 33% (67/203) of the sample identified as Black or African American, and approximately 8.9% (18/203) identified as Hispanic or Latina. At the prenatal visit, 90.1% (183/203) of mothers were currently in a romantic relationship, 66.9% (136/203) were married to their partner, and 84.06% (170/20,223) were living with their partner, defined as spending a minimum of 3 nights together at the same residence).

### Design and Procedures

#### Overview

The COVID-19 pandemic had 3 impacts on the design and execution of the BEE study. First, the pandemic interrupted and prolonged study recruitment procedures resulting in a recruitment window of 26 months. Second, some of the in-person planned visits were adapted to be conducted on the web and involve family-led data collection activities. This was most disruptive during the prenatal, 2-week, and 6-month visits that had already begun as *in-person* protocols and were shifted to web-based protocols because of the COVID-19 pandemic. Third, the timings of some visits were delayed. Although the original plan included 15- and 24-month visits, the final data collection timeline shifted these visits to 18 and 30 months. Each of these issues is discussed in the context of a brief synopsis of the overall study timeline.

Following recruitment, pregnant women are seen at a prenatal laboratory-based visit. Research staff follow up with participants around the expected due dates to speak with the participant as soon as possible following the child’s birth. During this conversation, staff determines if any new exclusion criteria for participation in the study are present—this includes being born with low birth weight (<2500 grams) or a gestational age below 36 weeks and 4 days, being in the neonatal intensive care unit for >24 hours after birth, being on a ventilator at birth, having had surgery or a chronic illness that would prevent participation, or demonstrating a medical reason that would prevent them from entering the fMRI scanner (eg, cochlear implants or other metal in the body). Exclusion criteria are consistent with our emphasis on the influence of poverty on individual differences in typically developing children. Those retained in the study are scheduled for a laboratory visit at 2 weeks postpartum. Participants are next seen during a home visit at 6 months of age (some were seen only via video-based visits on the web because of the COVID-19 pandemic) and then on a video-based visit on the web at 18 months of age (because of the COVID-19 pandemic). Next, participants are seen for a video-based visit on the web at 30 months of age, followed immediately by a laboratory visit also at 30 months of age (for the second fMRI scan). Finally, participants are seen at the laboratory for their last data collection visit at 36 months of age.

#### Prenatal Laboratory Visit (and Web-Based Visit During the COVID-19 Pandemic)

Pregnant women were invited to participate in their first data collection visit for the BEE study at the Biobehavioral Laboratory at the University of North Carolina (UNC) at Chapel Hill School of Nursing. Following a description of the overall study and the prenatal data collection protocol, informed consent for participation was obtained. Next, participants were interviewed by research staff, completed a series of questionnaires, a series of computer-based cognitive assessment tasks, and engaged in a qualitative interview about their experiences during pregnancy and expectations about motherhood. Finally, the research staff collected hair, blood, saliva, and urine samples that were immediately processed and placed in cold storage at −80 °C. Participants were compensated for their travel costs and provided US $50 for their time. For this visit protocol, some visits were conducted in person in the laboratory, and others were done via Zoom call because of the human participants contact restrictions related to the COVID-19 protocols. The same tasks and instructions are used in each visit, but the computer-based cognitive assessment tasks and the blood collections were no longer possible in the web-based protocol. For participants who needed internet or computing devices for the Zoom call, we provide tablets and wireless hot spots to facilitate the web-based visit. For those receiving the web-based protocol, biospecimen collection materials were mailed to the home, and participants returned the materials to the study via mail.

#### The 2-Week Laboratory Visit (and Web-Based Visit During the COVID-19 Pandemic)

Participant mothers and children visited the UNC Biomedical Research Imaging Center for the 2-week laboratory visit. After providing informed consent for the visit, mothers complete a short series of questionnaires, and infants are fed, swaddled, and rocked or held comfortably by the mother until they fall asleep. Parents apply ear protection when they determine it will be most tolerable for their child. The brain scanning occurs during natural sleep using a Food and Drug Administration–approved 3 Tesla fMRI scanner and under continuous monitoring by study personnel. A pulse oximeter is placed on the child’s toe to monitor oxygen levels and heart rate. Parents who complete an fMRI safety form are also allowed into the scanner suite with their child. Participants were compensated for their travel costs and provided US $100 for their time. For this visit protocol, some visits were conducted in person in the laboratory, and others were done via Zoom call because of the human participants contact restrictions related to the COVID-19 protocols. For web-based protocol participants, no fMRI scan was completed; only questionnaire data were collected.

#### The 6-Month Home Visit (and Web-Based Visit During the COVID-19 Pandemic)

Research staff visit participants in their homes for the 6-month home visit. After providing informed consent for the visit, mothers answered interview items and completed a series of questionnaires, and then the mother and child participated in 2 parent–child interaction tasks. The first involves playing together with a standardized set of toys for 10 minutes; the second involves the mother going through a wordless picture book with her child for 5 minutes. Both tasks are video and audio recorded for later behavioral coding. After these tasks, the research staff explain the extended data collection protocols for child sleep hygiene (7 days) and language exposure (2 days) after the visit. To collect sleep hygiene data, mothers are provided with a wearable actigraphy device (Actiwatch-2) for the child and instructed on how to attach it to the infant’s ankle. Mothers are asked to leave the device on the child’s ankle for 7 full days, remove it only during bath times, and then reattach it once the child is dry. Mothers are also provided a sleep diary to complete every morning for the next 7 days. To collect language exposure data, mothers were provided with a Language Environment Analysis (LENA) recording device, 2 vests, and 2 daily diaries. Mothers are asked to identify 2 typical days (out of the next 7) in which they will be spending most of their time with their child. On those days, mothers are asked to dress their child as they normally would first thing in the morning, then turn on the LENA recorder and place it in the pocket of the vest before putting the vest on the child. Mothers are asked to leave the device on all day. These steps are repeated for another day during the 7-day period. Mothers are also asked to complete daily diaries about the contexts and people in the child’s environment during those days. Both the actigraphy and the LENA devices are retrieved from the participant’s home on the seventh day following the 6-month home visit. Participants were compensated for their travel costs and provided US $50 for their time.

For this visit protocol, some visits were conducted in person in the participant’s home, and others were done via Zoom call because of the human participants contact restrictions related to the COVID-19 protocols. The same tasks and instructions are used in each visit, but the parent–child interaction tasks were recorded using Zoom instead of in-person cameras. For participants who needed internet or computing devices for the Zoom call, we provide tablets and wireless hot spots to facilitate the web-based visit. For those receiving the web-based protocol, wearable devices and biospecimen collection materials were mailed to the home, and participants returned the materials to the study via mail.

#### The 18-Month Remote Visit

The research staff invited participants to join a video call for the 18-month Zoom visit. After providing informed consent (electronically) for the visit, mothers answer interview items and complete a series of web-based questionnaires. Wearable devices and biospecimen collection materials were mailed to the home, and participants returned the materials to the study via mail. Participants were compensated US $50 for their time.

#### The 30-Month Remote Visit

The research staff invited participants to join a video call for the 30-month Zoom visit. After providing informed consent (electronically) for the visit, mothers complete a series of questionnaires, and then mother and child participate in a parent–child interaction task. This task involves presenting the child with a series of 3 peg puzzles of increasing difficulty and instructing the mother that she can provide any assistance that she chooses. The task is video and audio recorded for later behavioral coding. The research staff explain the extended data collection protocols for child sleep hygiene (7 days) and language exposure (2 days) that continue after the visit. Additional components of this study visit are identical to those protocols used in the 6-month home visit described earlier (eg, actigraphy, LENA, and biomarkers). Wearable devices and biospecimen collection materials were mailed to the home, and participants returned the materials to the study via mail. Participants were provided US $50 for their time.

#### The 30-Month Laboratory Visit

Participant mothers and children came to the UNC Biomedical Research Imaging Center for the 30-month laboratory visit for their second fMRI scan. As at the 2-week visit, this scan occurs while children are naturally sleeping. To facilitate this, the 30-month visits are scheduled during the evening at the child’s usual bedtime. After providing informed consent for the visit, mothers complete a short series of questionnaires, and children’s natural bedtime routine is replicated to facilitate child sleep. Next, the research staff administer the Receptive Vocabulary, Information, Block Design, and Object Assembly subscales of the Wechsler Preschool and Primary Scale of Intelligence (the former 2 subscales index vocabulary acquisition and the latter 2 index visual-spatial abilities) [50]. Mothers apply ear protection when they determine it will be most tolerable for their child. At this point, the research staff follow the same fMRI protocol as used during the 2-week laboratory visit. Participants were compensated for their travel costs and provided US $125 for their time.

#### The 36-Month Laboratory Visit

Participant mothers and children came to the Biobehavioral Laboratory at the Frank Porter Graham Child Development Center at UNC Chapel Hill for the 36-month laboratory visit. After providing informed consent for the visit, mothers answer interview items and complete a series of questionnaires. During this time, research staff assess children’s EF using the EF Touch battery [51] and assess children’s language using the Peabody Picture Vocabulary Test [52]. Participants were compensated for their travel costs and provided US $50 for their time.

### Measures

A list of all interviews and questionnaires administered at each data collection visit is provided in Multimedia Appendix 2. In the further sections, we describe the measurement of each key construct used to address the specific aims 1, 2, and 3 in greater detail.

#### Household Income

At each data collection visit, mothers reported (1) their annual income, (2) the annual income of their partner (if co-residing in the home), (3) the annualized contributions to the household of all others in the household, and (4) the annualized contributions from *other* sources of income (eg, unemployment insurance, worker’s compensation, and social security retirement). Using this information, an annual household total income variable is created by summing all sources of income. Next, we divide this total amount by the federal poverty threshold for a family of that particular size and composition to create the income-to-needs (ITN) ratio—a standard measure of a family’s economic situation where a value of 1.0 indicates living right at the poverty line. This approach to quantifying household income has been used extensively in population-based studies of poverty and child development [49].

#### Prenatal Stress

##### Self-report of Subjective Stress Experience

In all, 3 self-report questionnaires on experiences of stress are completed at the prenatal visit. The Cohen Perceived Stress Scale is a 10-item questionnaire measuring *general perceived stress* during the past month [53]. The items are rated on a 5-point Likert scale ranging from *never* (0) to *very often* (4) and have demonstrated high reliability; the reliability of Perceived Stress Scale in this study is 0.88. The Pregnancy-Related Anxiety Questionnaire–Revised-2 is a 10-item measure assessing *pregnancy-related stress* [54]. The items are rated on a 5-point Likert scale ranging from 0=*definitely not a concern at all* to 4=*definitely a very big concern*. The reliability of this scale in this study is 0.85. A modified 6-item version of the Economic Strain Questionnaire measures *financial stress*, including concerns about the inability of families to *make ends meet* and not having enough money for a home, clothing, food, and medical care [55]. Items are rated on a 5-point Likert scale (0=great deal of difficulty to 4=no difficulty at all) and on a 4-point Likert scale (0=strongly disagree to 3=strongly agree). Previous studies report adequate reliability of this scale and suggested that all 6 items could be standardized and summed or averaged to create a global measure of economic strain [55,56]. The reliability of this scale in this study is 0.88.

##### Biological Indexes of Prenatal Stress

A total of 2 classes of biological measures of stress were collected during the prenatal visit. The first includes hair cortisol concentration measured from hair strands close to the scalp from the posterior vertex area of the participant’s head (this area has shown the lowest coefficient of variation [CV]). The 3 cm of hair closest to the hair roots are analyzed [57], reflecting exposure during the last 3 months (based on the hair growth rate of approximately 1 centimeter per month) [58]. Cortisol is measured in methanol extracts of hair using a competitive radioimmunoassay following standard assay procedures. The intra-assay CV is 1.17%, and the interassay CV is 5.12% in this sample—both values indicate reliable assaying results.

The second class of biological stress indexes includes multiple inflammatory markers observed during the prenatal visit. A total of 2 EDTA tubes are used to conduct antecubital venipuncture to collect nonfasting plasma samples from participants. Plasma samples are assayed for cytokines using a Meso Scale Discovery multiplex kit; this panel includes interleukin-6, interleukin-10, tumor necrosis factor, interleukin-1RA, interleukin-2, and interleukin-8, each measured in pg/mL. C-reactive protein is assayed using Meso Scale Discovery single-plex kit and is measured in mg/L.

#### Early Postnatal Experience

##### Caregiving

Video recordings of parent–child interactions will be coded by trained and certified coders to rate caregiving behaviors on the following dimensions: sensitivity, intrusiveness, detachment, stimulation of cognitive development, positive regard, negative regard, and animation. *Sensitivity* indexes the degree to which the caregiver is attuned and responsive to the physical and emotional needs of the child. *Intrusiveness* indexes the degree to which the caregiver is controlling and imposes their own agenda on the activity of the child. *Detachment* indexes the degree to which the caregiver is emotionally and physically detached and uninvolved with the feelings and activities of the child. *Stimulation of cognitive development* indexes the degree to which the caregiver provides linguistic stimulation in a developmentally appropriate way and scaffolds the activity to maximize the child’s cognitive experiences of the task. *Positive regard* indexes the degree to which the caregiver directs feelings of warmth, love, and enjoyment toward the child. *Negative regard* indexes the degree to which the caregiver directly displays harshness and hostility toward the child. *Animation* indexes the level of energy and enthusiasm that the caregiver displays while interacting with the child. Each dimension is rated on a 7-point scale ranging from *not at all characteristic of this caregiver* to *extremely characteristic of the caregiver*. After being certified as reliable, each coder will continue to code a minimum of 20% of cases with a master coder for each coding assignment to prevent coder drift. Previous use of this parent–child interaction task and coding protocol have repeatedly identified 2 parenting composites guided by factor analyses [24,59], and we anticipate observing comparable factors. The first is referred to as *sensitivity* and is the mean of sensitivity, detachment (reversed), stimulation of cognitive development, positive regard, and animation. The second is *harsh intrusiveness* and is the mean of intrusiveness and negative regard.

##### Sleep Hygiene

A multi-method approach will be applied to assess sleep hygiene [60,61]. The actigraphy monitor worn on the child’s ankle contains an accelerometer that measures limb movement in 15-second epochs. At the end of the sleep assessment week, actigraphy data are downloaded to a computer and edited using Phillips Actiware software (version 6.0). The Actogram algorithm settings are as follows: immobile minutes for sleep onset were set to 5 minutes; minimum rest interval size was set to 20 minutes; multiple rest intervals per day were allowed; automatically set minor rest intervals were allowed. The activity threshold for scoring the child as awake is set to the automatic setting (0.888×average activity count)—both the algorithm and threshold for scoring sleep or wake state have been previously validated [62].

The output from the Actiware program includes a listing of all sleep and wake intervals. Infant sleep onset time is determined as the start time of the sleep interval closest to the caregiver-reported bedtime (see sleep diary description below). Similarly, the child’s rise time is determined as the end time of the sleep interval closest to the caregiver-reported rise time. Using sleep onset time and rise time, we subsequently calculate the duration of the child’s sleep period. Child sleep time in minutes is determined by summing infant sleep time in each sleep interval between sleep onset time and rise time. Child wake time in minutes is determined by summing infant wake time in each sleep interval during the sleep period. Child night wakings are determined by subtracting 1 from the number of sleep intervals during the nighttime sleep period (ie, if the child sleeps in 3 sleep intervals, there are 2 night wakings). Finally, the longest sleep period equals the duration of the longest sleep interval during the nighttime sleep period.

In addition to actigraphy measures, the parent completes daily sleep diaries for the child. Every day during the sleep assessment week, research staff call mothers to obtain information about the previous day’s naps and nighttime sleep, including number, location, and duration of naps, child bedtime, number of night wakings, types of interventions used during night wakings, and infant rise time [63]. Mothers also report any unusual occurrences that may have influenced the previous night’s sleep, such as child illness.

##### Language Exposure

The LENA digital recorder and software automatically processes the audio-based language environment the child experiences during the 2 days of data collection. As a wearable device, the LENA recorder collects recordings of the language environment for the entire day (16 hours; for the purposes of this study, wake time to bedtime). After 2 full days of data collection, the LENA recorder connects to a computer, and its software automatically uploads and analyzes the language data using a series of iterative modeling algorithms developed by the LENA Research Foundation. This process segments the recordings based on acoustic energy and generates 3 language measures mapping onto the Hart and Risley language exposure dimensions—adult word, conversational turn, and child vocalization counts [64]. Adult word count is the total number of adult words spoken near the child. Conversational turn count is the total number of conversational interactions the child engages in with an adult (this involves one person speaking and the other responding within 5 seconds). Child vocalization count is the total number of speech-like utterances produced by a child. LENA software also generates other language indexes, such as overlapping speech, television and media, and background noise. This study focuses on adult word count and conversation turns as key indicators of child language exposure in the first 3 years of life.

#### Brain Development

DTI and rs fMRI scans are acquired at 2 weeks and 30 months of age. Scanning sequences are fully compatible with the Human Connectome Project (Multimedia Appendix 3). We use previously established image processing pipelines, quality control measures, and analysis protocols, which have proven successful for repeat scanning with infants and young children [65-68].

##### DTI Analysis

Diffusion images are screened using an automatic program for quantifying motion artifacts and corrupted sections using the diffusion-weighted imaging or DTI analysis quality control tool DTIPrep [69]. This program includes the correction of motion, eddy of current artifacts, and removal of outliers. Diffusivity property maps, such as fractional anisotropies, are estimated using standard weighted least square fitting [70]. The Brain Extraction Tool [71] is used for the skull stripping of all images. Unbiased atlas building [70] with large deformation diffeomorphic metric mapping registration [72] is used after a linear registration [73]. All data to be analyzed in this study are used to build the atlas. Transformations obtained from the atlas building are applied to warp the original tensor images to the atlas space, and the final DTI atlas is obtained by averaging the warped images. DTI tractography in atlas space yields all fiber bundles of interest [74].

Our established tractography pipelines generate quantitative DTI data for cohesive analysis of imaging data collected at 2 weeks and 30 months of age. Diffusion properties (fractional anisotropy, axial diffusivity, and radial diffusivity) are generated for white matter tracts hypothesized to support emerging EFs and language, including arcuate, uncinate, and anterior cingulum. Tractography algorithms with Runge-Kutta integration are performed in the atlas tensor image constructed from the unbiased DTI registration. The improved signal-to-noise ratio of participant-specific atlases (integrating data from 2 weeks to 30 months), deformed to a group atlas, allows reliable extraction of fiber bundles that would be hard to extract consistently from individual data (ie, arcuate). Fiber tracts are parameterized by length to represent diffusion properties as a function of location along the selected tracts [73,74].

##### Resting State

We will primarily use a seed-based approach to characterize the dynamic developmental changes of functional networks related to attention, salience, executive control, and default mode. Specifically, network-specific seeds will be defined according to our previous studies and used to generate functional connectivity maps of each network for the scans collected at 2 weeks and 30 months. Two measures of network integrity will be derived: mean functional connectivity and network maturation score. For the former, after defining the functional connectivity map of each network, individual clusters of significance will be extracted, and a cross-correlation matrix among all clusters within the same network will be calculated for each participant. The mean functional connectivity will be defined as the average of all pair-wise correlations within the network. This measure quantifies the individual functional network integrity in an age-adaptive fashion based on age-specific functional connectivity maps. For our measure of network maturation score, we will calculate a network maturation score using adult functional network topology as a reference to quantify the degree of maturation toward adult-like network topology [75]. On the basis of the same adult reference, this measure facilitates statistical comparisons across age groups and has been shown to be sensitive to SES during infancy [76]. Specifically, using the adult group-level significant functional networks as references, a binary mask was derived for each network. Subsequently, the within-network connectivity was defined as the mean functional connectivity strength within the mask, indicating the degree of within-network synchronization, whereas outside-network connectivity was the mean functional connectivity of areas outside the network mask, indicating the degree of outside-network specialization. Finally, based on a previously established network matching concept [77], the subtraction of the outside-network connectivity from within-network connectivity yields a network matching score, indicating the degree of similarity between the network in question and the adult reference network in terms of functional connectivity strength distribution of the whole brain. This network matching score will be used as an overall measure to quantify the maturation of individual networks.

To test the robustness of our results, a data-driven independent component analysis approach [78], which has been used extensively in previous studies, will be applied to extract the corresponding networks and subsequent network-level functional connectivity measures [79]. Identical analysis as listed above will be carried out based on independent component analysis–based measures to replicate our findings using a seed-based approach. Finally, given the controversy of the preprocessing step of global signal regression [80,81], our results will also be tested with or without this step to identify converging findings.

##### EF Skills

Children’s EF is measured at 36 months using the EF Touch, a computerized battery of EF tasks that have been iteratively developed over the last 10 years [82-84]. Each EF task takes 3 to 7 minutes to complete. A total of 2 *warm-up* tasks are typically administered first to acclimate children to using the touch screen. The reliability and validity of the EF Touch battery have been extensively documented, including with children aged as young as 3 years [85]. Mean accuracy across items within each task will index task performance.

Three tasks assess children’s *inhibitory control*: *Spatial Conflict Arrows*, *Silly Sounds Stroop*, and *Animal Go/No-Go*. Two tasks will assess children’s *working memory* at 36 months of age: *Working Memory Span* and *Pick a Picture*. One task will assess children’s *attention shifting* at 36 months of age: *Something’s the Same*. In addition to performance-based measures, assessors will complete the Preschool Self-Regulation Assessment following the administration of tasks. The Preschool Self-Regulation Assessment consists of 28 items that are combined to form attentional control and positivity scales that reflect behavior during EF task completion [86].

### Analysis Plan for Key Aims

Before conducting statistical analyses, the psychometric properties (ie, item to total correlations and α coefficients) of all questionnaires will be evaluated, and the interrater reliability (ie, intraclass correlations and α or κ coefficients) of measures that were based on observational coding will be documented. Descriptive statistics will be computed for all measures with an emphasis on distributions and outliers. Data transformations will be considered for variables appreciably skewed. When multiple measures are correlated within an assessment period (eg, caregiver-reported stressors during the prenatal visit), principal components and factor analyses will be used to create composite scores.

A structural equation modeling approach will be used to test all study aims. All structural equation modeling models will be estimated using a robust full information maximum likelihood estimator. The robust full information maximum likelihood estimator accommodates nonnormally distributed outcome variables and represents a statistical best practice for accommodating missing or unbalanced data [87,88]. To maximize power and avoid distributional assumptions, bootstrapped tests of indirect effects will be used to test questions of mediation related to aims 2 and 3 [89,90]. An exemplar path diagram corresponding to aims 1 to 3 is depicted in Figure 1 (residual variances or covariances are omitted to simplify the presentation).

Examination of direct and indirect effects will provide formal tests of aim 1. This will include testing a direct effect of prenatal poverty on brain development at 2 weeks of age and testing this path as an indirect effect mediated by subjective measures of prenatal stress (from self-report) and biological indexes of stress (indexed by hair cortisol and inflammatory markers).

Examination of direct and indirect effects will provide formal tests of aim 2. Direct effects from postnatal poverty (aggregated across 6, 18, and 30 months of age) on brain development at 30 months (controlling for comparable neurological indexes at 2 weeks of age) will test the association between poverty and brain development in the first 30 months of life. We will evaluate a measurement model for a latent caregiving environment variable based on observed caregiving, sleep hygiene, and language exposure at 6 and 30 months of age. This latent variable will be examined as a partial mediator in the indirect path from postnatal poverty to brain development at 30 months of age. If the measurement model fails to establish an adequate fit, we will use each individual’s indicators of early life experience as predictors or mediators in the model.

Each of the models described for testing hypotheses in aims 1 and 2 will be extended to include an EF composite, measured at 36 months, as a distal outcome. The EF composite will be regressed onto the 2-week and 30-month parameters for each DTI and rs metric, caregiving environment latent variable, prenatal stress measure, and prenatal and postnatal indicators of poverty (if prenatal and postnatal indicators of poverty are collinear, a single combined index will be used). The tests of direct and indirect effects of prenatal and postnatal poverty will provide a formal test of aim 3.

The previous analyses will provide definitive tests of all study aims. However, as described in the *Measures* section, we will also collect multiple indicators of general cognitive development (eg, Wechsler Preschool and Primary Scale of Intelligence and expressive language) at earlier assessments, and these interim measures will be used as outcomes in analyses that begin to test the guiding questions of this study before the completion of final data collection. These analyses will consist of simplified variations of the models described above. Interim measures will also be used for sample description.

### Ethical Considerations

The study has institutional review board approval from the University of North Carolina at Chapel Hill (study number 17-1914).

## Results

**Figure 2:**
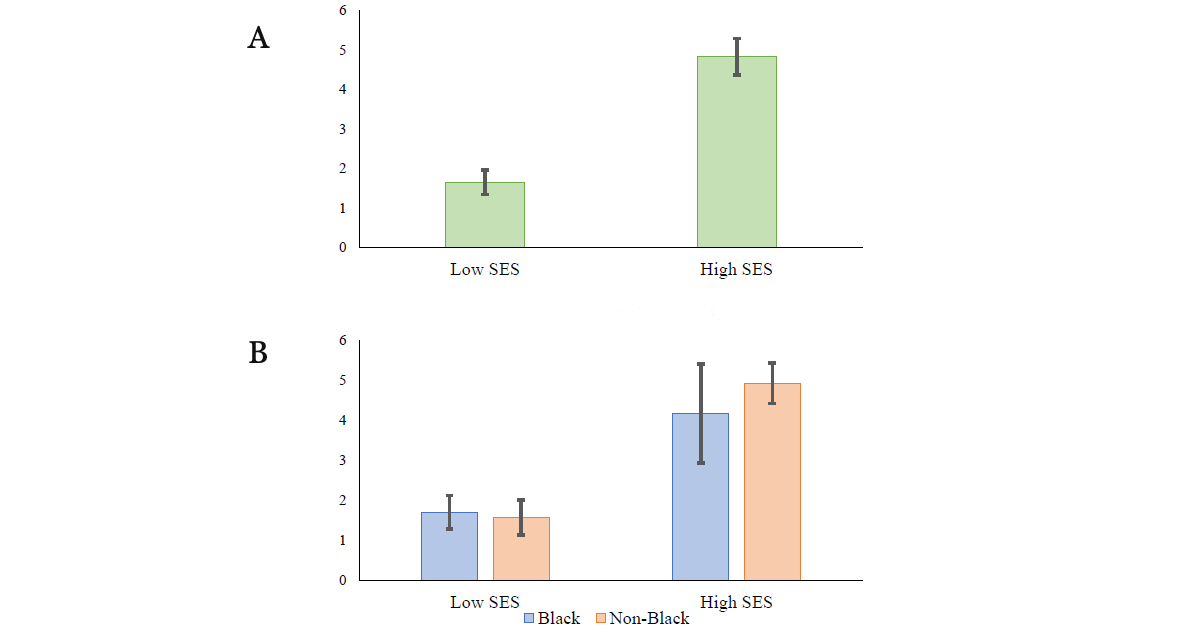
Means and SEs (−2 to 2) for income-to-needs ratios across recruitment cells. (A) Socioeconomic status (SES)-only cells and (B) level and SES×race cells.

Recruitment is complete with a sample of 203 participants. Data collection began in September of 2018 and is expected to conclude by February 2024. This is a prospective longitudinal study; thus, analyses addressing the project’s specific aims are not yet available. However, analyses addressing the validity of the project’s recruitment strategy are provided by examination of the distribution of ITN ratios (a ratio of annual household income relative to a federal poverty threshold for a given household size, as described in the *Measures* section), the amount of money immediately available in savings or checking accounts, and homeownership data (all reported at the prenatal visit). We identified a participant as an outlier with an ITN ratio of 37.88; to limit the effects of this extreme value, we winsorized it to the next highest value of 15.79. The ITN distribution remained positively skewed with a minimum value of 0.00 and a maximum value of 15.79 (see Multimedia Appendix 4 for descriptive ITN information for the entire sample and separately by recruitment cell). For the total sample, 32.5% (66/203) of the participants reported an ITN ratio of <2.0 (interpreted as *near poor* or *working poor*), and 13.3% (27/203) of the participants reported an ITN ratio of <1 (interpreted as *poor* per the US federal definition of poverty). Next, we examined ITN distributions by recruitment SES designation. Of those recruited as *low SES*, 71% (55/78) reported ITN ratios of <2.0, and 35% (27/78) reported ITN ratios of <1.0. Among participants recruited into the *not-low SES* stratum, only 8.8% (11/125) reported ITN ratios of <2.0, and no participant reported ITN ratios of <1.0. The average ITN ratio for participants recruited into the low-income stratum was significantly lower than the average for the high-income recruitment cell (*t*_188_=−9.643; *P*<.001; Figure 2).

Comparable percentages of income levels across SES designations at recruitment were observed for Black and non-Black participants. As seen in Figure 2, within racial categories, (1) non-Black low-income participants reported lower ITN ratios than non-Black high-income participants (*P*<.001), and (2) Black low-income participants reported lower ITN ratios than Black high-income participants (*P*=.002). Within SES categories, (1) non-Black low-income participants were not statistically different from Black low-income participants, and (2) non-Black high-income participants were not statistically different from Black high-income participants.

**Figure 3:**
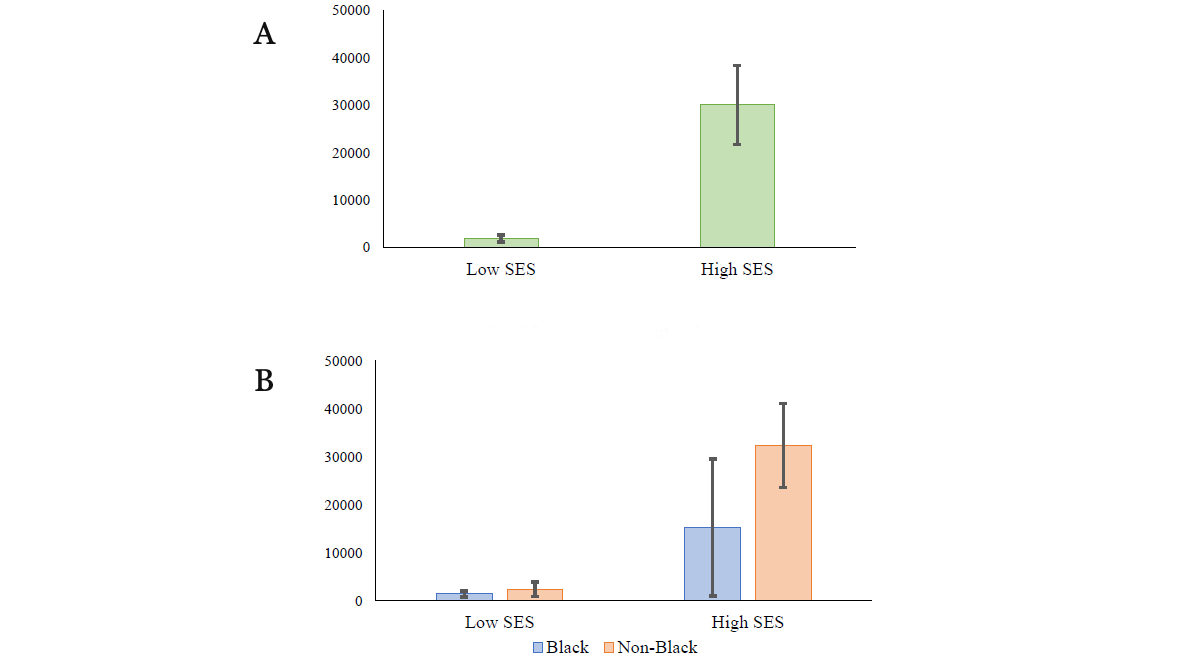
Means and SEs (−2 to 2) for bank savings across recruitment cells. (A) Socioeconomic status (SES)-only cells and (B) level and SES×race cells.

Although ITN is a broad indicator of a family’s financial standing, another indicator of economic insecurity is the availability of liquid funds, such as money in bank savings or checking accounts. According to the US Federal Reserve, in 2018, only 61% of Americans had immediate access to funds to cover a US $400 emergency expense [91]. In the study sample participating in this study, there are similar distributions and mean differences for participant reports of money immediately accessible in bank savings and checking accounts (Multimedia Appendix 5) compared with household ITN ratios. The average bank savings for the low-income recruitment cell was significantly lower than the average for the high-income recruitment cell (*t*_181_=−5.44; *P*<.001; Figure 3).

Although ITN and immediately accessible funds are important indicators of economic security, individual differences among families in overall wealth may also differentially buffer families from stress and negative life events (and racial disparities in wealth have been studied less than income despite being potentially larger in magnitude). An indicator of family wealth is homeownership. Among those recruited as high-SES, 76.8% (96/125) owned their homes compared with 20.8% (26/125) that rented their homes (note that these percentages do not sum to 100% because some participants lived with family and thus neither owned nor rented their homes). Conversely, among participants recruited as low-SES, only 8% (6/78) owned their homes, whereas 82% (64/78) rented their homes. Rates of homeownership for non-Black participants were similar to the overall sample. However, for Black participants, homeownership rates were lower. For high-SES Black families, only 35% (6/17) owned their own homes; for low-SES Black families, only 6% (3/48) owned their homes.

In summary, across these 3 indicators (ITN ratio, bank savings, and homeownership), there is convergent evidence validating the recruitment strategy’s goal of establishing a sample that is (1) economically diverse with adequate representation of low and very low-income participants and (2) economically diverse within Black and non-Black subsamples to reduce potential confounding between race and income.

## Discussion

The BEE study is one of the first prospective longitudinal studies of the prenatal and postnatal environmental influences on emerging EF in the first 3 years of life with specific foci on the potential effects of poverty, poverty-related stressors, and neurological substrates underlying cognitive abilities. Three primary aims examine (1) the associations between objective poverty and subjective prenatal experience on prenatal structural and functional brain development, (2) the associations between objective poverty and subjective postnatal experience on structural and functional brain development during the first 2.5 years of life, and (3) the associations between objective poverty and subjective postnatal experience on EF at 3 years of life. As described in this paper, several accommodations to this study were made in response to the COVID-19 pandemic, including prolonged participant recruitment, shifts to web-based data collection for some measurements, and alternation in the timing of visits and assessments. These accommodations have minimized the potential threats posed by the COVID-19 pandemic to the internal validity of the study. Despite the prolonged recruitment period, the results support the validity of the overall recruitment strategy, which was designed to establish a socioeconomically diverse sample with adequate variability in family incomes within both Black and non-Black families to reduce the potential confound of race and income. The web-based data collection of questionnaire data and observational protocols and the remote-based data collection of language exposure and child sleep hygiene have resulted in data comparable (qualitatively and quantitatively) with in-person data collection protocols. Finally, the changes in the timing of data collection visits maximized the number of participants that could participate in fMRI data collection while remaining within the targeted developmental window for examining neurological development pertinent to emerging EF within the first 3 years of life. We believe that the data being collected and subsequent analyses addressing the specific aims of this study will not be compromised by the COVID-19 pandemic.

## Appendix

Multimedia Appendix 1 Recruitment flowchart leading to the sample of 203 participants.

Multimedia Appendix 2 Questionnaires administered across data collection visits.

Multimedia Appendix 3 Magnetic resonance imaging acquisition and preprocessing methods.

Multimedia Appendix 4 Descriptive statistics for income-to-needs ratios for the total sample and stratified by recruitment cell.

Multimedia Appendix 5 Descriptive statistics for bank savings for the total sample and stratified by recruitment cell.

Multimedia Appendix 6 Peer-review report from the National Institute of Child Health and Human Development - Psychosocial Development, Risk and Prevention (PDRP) Study Section - Risk, Prevention and Health Behavior Integrated Review Group - Center for Scientific Review (National Institutes of Health, USA).

## Abbreviations

BEE: Brain and Early Experience
CV: coefficient of variation
DTI: diffusion tensor imaging
EF: executive function
fMRI: functional magnetic resonance imaging
ITN: income-to-needs
LENA: Language Environment Analysis
rs: resting state
SES: socioeconomic status
UNC: University of North Carolina
