# Supplementary material for: The Brain and Early Experience Study: Protocol for a Prospective Observational Study
Source: JMIR Res Protoc. 2022 Jun 29;11(6):e34854. doi: 10.2196/34854 (PMC9280455; doi:10.2196/34854)
Supplement: Multimedia Appendix 1 [file resprot_v11i6e34854_app1.docx]

**Multimedia Appendix 1.**

*Recruitment Flowchart Leading to N=203 Sample.*

**N = 3267**

Potential participants from online ads, clinic visits, or medical records

*N = 2559 not contacted for phone call*:

- Inclusion criteria not met (N=2295)
- Unable to reach by phone (N=264)

**N= 708**

Potential participants receiving

recruitment phone call

*N = 505 not eligible*:

- Not interested (N= 434)
- Unable to schedule visit (N= 53)
- No longer eligible (N=18)

**N = 203**

Formally enrolled with two of the first three data collection visits completed
