# Supplementary material for: The Brain and Early Experience Study: Protocol for a Prospective Observational Study
Source: JMIR Res Protoc. 2022 Jun 29;11(6):e34854. doi: 10.2196/34854 (PMC9280455; doi:10.2196/34854)
Supplement: Multimedia Appendix 2 [file resprot_v11i6e34854_app2.docx]

| **Multimedia Appendix 2.**  *Select Questionnaires Administered across Data Collection Visits* | | | | | | | |
| --- | --- | --- | --- | --- | --- | --- | --- |
| Construct | Measure | Pre-natal | 2-wk | 6-mo | 18-mo | 30-mo | 36-mo |
| Household composition | BEE Generated Measure | ✓ | ✓ | ✓ | ✓ | ✓ | ✓ |
| Income | BEE Generated Measure | ✓ | ✓ | ✓ | ✓ | ✓ | ✓ |
| Health & Medication Use | BEE Generated Measure | ✓ | ✓ | ✓ | ✓ | ✓ | ✓ |
| Parent Stress – Financial | Economic Strain Questionnaire | ✓ |  | ✓ |  | ✓ |  |
| Parent Stress – General | Cohen Perceived Stress Scale | ✓ |  |  |  |  |  |
| Parent Stress – Pregnancy | Pregnancy Related Anxiety Scale | ✓ |  |  |  |  |  |
| Parent Stress – Racism | Race-Related Stressful Events | ✓ |  | ✓ | ✓ |  |  |
| Parent Stress – Racism | Experiences of Racism Scale | ✓ |  |  | ✓ |  |  |
| Parent Stress – Parenting | Parent Stress Index |  |  | ✓ | ✓ | ✓ |  |
| Intimate Partner Violence | Conflict Tactics Scale | ✓ |  | ✓ |  | ✓ |  |
| Social Support | Questionnaire of Social Support | ✓ |  | ✓ |  | ✓ |  |
| Personality | Ten-Item Personality Inventory | ✓ |  |  |  |  |  |
| Sleep Quality | Pittsburgh Sleep Quality Index | ✓ |  | ✓ |  | ✓ |  |
| Sleep Quality | Infant Sleep Questionnaire |  | ✓ |  |  | ✓ |  |
| Child Nutrition | Infant Feeding Questionnaire |  | ✓ | ✓ | ✓ |  |  |
| Quality of Home Env. | Stimq |  |  | ✓ |  | ✓ |  |
| Mental Health | Brief Symptom Inventory | ✓ |  | ✓ | ✓ | ✓ | ✓ |
| Mental Health | WHO Questionnaire (Adult ADHD) | ✓ |  |  |  |  |  |
| Mental Health | Barkley's Quick-Check (Adult ADHD) | ✓ |  |  |  |  |  |
| Temperament | Infant Behavior Questionnaire |  |  | ✓ |  |  |  |
| Temperament | Early Child Behavior Questionnaire |  |  |  |  | ✓ |  |
| Motor Ability | Early Motor Questionnaire |  |  | ✓ | ✓ | ✓ | ✓ |
| Language Ability | Macarthur Bates Comm Inv |  |  |  | ✓ | ✓ | ✓ |
| Internalizing/Externalizing | AESEBA: Child Behavior Checklist |  |  |  |  |  | ✓ |
| Covid-19 Experience | Covid-19 Questionnaire |  | ✓ | ✓ | ✓ | ✓ | ✓ |
