# Supplementary material for: The Brain and Early Experience Study: Protocol for a Prospective Observational Study
Source: JMIR Res Protoc. 2022 Jun 29;11(6):e34854. doi: 10.2196/34854 (PMC9280455; doi:10.2196/34854)
Supplement: Multimedia Appendix 3 [file resprot_v11i6e34854_app3.docx]

| **Multimedia Appendix 3**  *MRI Acquisition and Pre/Processing Methods* | | | | | | |  |
| --- | --- | --- | --- | --- | --- | --- | --- |
|  | FOV | Res (mm3) | TE  (ms) | TR (ms)/TI | Slice  Orient. | Other | |
| Localizer |  |  |  |  |  |  | |
| MPRAGE | 256x256 | 0.8x0.8x0.8 | 2.24 | 2400/1060 | 208/Sag |  | |
| T2w SPC | 256x256 | 0.8x0.8x0.8 | 564 | 3200 | 208/Sag |  | |
| DTI-AP | 201x210 | 1.5x1.5x1.5 | 89.2 | 3222 | 92/Axial | B=1500 s/mm2 | |
| Field Map-AP | 208x208 | 2x2x2 | 58 | 7700 | 72/Axial |  | |
| Field Map-PA | 208x208 | 2x2x2 | 58 | 7700 | 72/Axial |  | |
| Resting BOLD-PA | 208x208 | 2x2x2 | 37 | 720 | 72/Axial | 420 measures | |
| Resting BOLD-AP | 208x208 | 2x2x2 | 37 | 720 | 72/Axial | measures | |
| *Notes*: Neuroimaging is conducted during natural sleep for all participants in this study. Abbreviations are as follows: MPRAGE = magnetization-prepared rapid gradient-echo; T2w SPC = T2 weighted space; PA = posterior to anterior; AP = anterior to posterior; DTI = diffusion tensor imaging; BOLD = blood oxygenation level dependent; FOV = field of view; TE = echo time; Res = resolution; TR = repetition time; TI = inversion time; Sag = sagittal. | | | | | | |  |
