# Supplementary material for: The Brain and Early Experience Study: Protocol for a Prospective Observational Study
Source: JMIR Res Protoc. 2022 Jun 29;11(6):e34854. doi: 10.2196/34854 (PMC9280455; doi:10.2196/34854)
Supplement: Multimedia Appendix 4 [file resprot_v11i6e34854_app4.docx]

| **Multimedia Appendix 4** | | | | | | | | | | |
| --- | --- | --- | --- | --- | --- | --- | --- | --- | --- | --- |
| *Descriptive Statistics for Income-To-Needs (ITN) Ratios for the Full Sample and as Stratified by Recruitment Cell* | | | | | | | | | | |
|  | Total Sample (n = 203) | | |  | Not Black (n = 138) | |  | Black (n = 65) | |  |
|  | Low  (n = 78) | High  (n = 125) | Total  (n = 203) |  | Low  (n = 30) | High  (n = 108) |  | Low  (n = 48) | High  (n = 17) |  |
| Mean | 1.65 | 4.83 | 3.59 |  | 1.57 | 4.93 |  | 1.70 | 4.18 |  |
| SD | 1.36 | 2.62 | 2.71 |  | 1.21 | 2.63 |  | 1.46 | 2.55 |  |
| Min | 0.00 | 1.50 | 0 |  | 0.29 | 1.54 |  | 0.00 | 1.50 |  |
| Max | 5.94 | 15.70 | 15.70 |  | 5.94 | 15.70 |  | 5.65 | 11.28 |  |
| % ITN < 1 | 34.6% | 0.00% | 13.3% |  | 32.1% | 0.00% |  | 34.8% | 0.00% |  |
| % ITN < 2 | 70.5% | 8.60% | 32.5% |  | 71.4% | 7.90% |  | 69.6% | 13.3% |  |
