# Supplementary material for: The Brain and Early Experience Study: Protocol for a Prospective Observational Study
Source: JMIR Res Protoc. 2022 Jun 29;11(6):e34854. doi: 10.2196/34854 (PMC9280455; doi:10.2196/34854)
Supplement: Multimedia Appendix 5 [file resprot_v11i6e34854_app5.docx]

| **Multimedia Appendix 5** | | | | | | | | | |
| --- | --- | --- | --- | --- | --- | --- | --- | --- | --- |
| *Descriptive Statistics for Bank Savings for the Full Sample and as Stratified by Recruitment Cell* | | | | | | | | | |
|  | Total Sample (n = 203) | | |  | Not Black (n = 138) | |  | Black (n = 65) | |
|  | Low  (n = 72) | High  (n = 111) | Total  (n = 183) |  | Low  (n = 30) | High  (n = 108) |  | Low  (n = 48) | High  (n = 17) |
| Mean | 1,877 | 30,076 | 18,981 |  | 2,456 | 32,385 |  | 1,487 | 15,296 |
| SD | 3,160 | 43,910 | 36,878 |  | 4,236 | 45,450 |  | 2,129 | 29,354 |
| Min | 0 | 0 | 0 |  | 0 | 0 |  | 0 | 0 |
| Max | 20,000 | 233,000 | 233,000 |  | 20,000 | 233,000 |  | 9,000 | 100,000 |
