# Supplementary material for: The Brain and Early Experience Study: Protocol for a Prospective Observational Study
Source: JMIR Res Protoc. 2022 Jun 29;11(6):e34854. doi: 10.2196/34854 (PMC9280455; doi:10.2196/34854)
Supplement: Multimedia Appendix 6 [file resprot_v11i6e34854_app6.pdf]

SUMMARY STATEMENT
(Privileged Communication)

PROGRAM CONTACT:
JAMES GRIFFIN
(301) 435-2307
griffinj@mail.nih.gov

Release Date: **06/19/2017**
Revised Date:

Application Number: **1 R01 HD091148-01A1**

Principal Investigators (Listed Alphabetically):

PROPPER, CATHI BARBRA (Contact)
SHORT, SARAH J

Applicant Organization: UNIV OF NORTH CAROLINA CHAPEL HILL

Review Group: **PDRP**
Psychosocial Development, Risk and Prevention Study Section

Meeting Date: **06/08/2017**
Council: **OCT 2017**
Requested Start: **12/01/2017**
RFA/PA: **PA16-160**
PCC: **CDBB -JG**

Project Title: **A mechanistic study of the association between poverty and executive functions in early childhood: Contributions of early brain development and the early caregiving environment**
SRG Action: **Impact Score:14 Percentile:2**
Next Steps: **Visit https://grants.nih.gov/grants/next_steps.htm**
Human Subjects: 30-Human subjects involved - Certified, no SRG concerns
Animal Subjects: 10-No live vertebrate animals involved for competing appl.
Gender: 1A-Both genders, scientifically acceptable
Minority: 1A-Minorities and non-minorities, scientifically acceptable
Children: 1A-Both Children and Adults, scientifically acceptable
Clinical Research - not NIH-defined Phase III Trial

Project Year	Direct Costs Requested	Estimated Total Cost
1	406,202	707,473
2	411,794	717,213
3	429,356	747,800
4	389,824	678,948
5	367,069	639,316
TOTAL	2,004,245	3,490,751

ADMINISTRATIVE BUDGET NOTE: The budget shown is the requested budget and has not been adjusted to reflect any recommendations made by reviewers. If an award is planned, the costs will be calculated by Institute grants management staff based on the recommendations outlined below in the COMMITTEE BUDGET RECOMMENDATIONS section.
NEW INVESTIGATOR

1R01HD091148-01A1 Propper, Cathi

NEW INVESTIGATOR

RESUME AND SUMMARY OF DISCUSSION: This application proposes to examine the link between poverty and executive functions that undergo developmental changes during the first years of life. The strong scientific premise to identify brain mediators through which poverty leads to poor executive functioning is based on the developmental health and disease model. This submission has been responsive to the previous review and the application has improved. The application innovation lies in the examination of poverty on executive functioning via the effects of neurological development during the early years of life. During the discussion, the reviewers noted that the rigorous comprehensive methods and measurement of executive functioning tasks, sleep language exposure, and parenting, the multimodal assessments of structural and functional neuroimaging, the strong power analyses, the impressive preliminary data and systematic recruitment procedures, and the planned missing design were strengths of the application. Overall, the committee agreed that the proposed research is highly significant and innovative and has the potential to have a high impact in enhancing understanding of the effects of prenatal and postnatal poverty on the development of the brain and executive functioning, as well as, identifying subsequent preventative and early intervention efforts.

DESCRIPTION (provided by applicant): Chronic stress for children growing up in poverty may lead to lasting effects on social, behavioral, and cognitive development. The difficulties of living in economic hardship has, indeed, been associated with deficits in cognitive and academic performance. The current study examines the link between poverty and executive functions (cognitive processes that facilitate learning, self-monitoring, and decision making) which are known to undergo rapid developmental change during the first years of life. Early neurological development will be examined as a mediator of this association examined from pregnancy to age 3. In addition to distal risk associated with living in poverty, we will investigate critical experiences within the proximal context (i.e., language exposure, caregiver behavior, child sleep hygiene) that may mediate the effect of this risk on child structural and functional brain development. Participants (n= 230) will be seen during the 28th week of pregnancy, and at 5 visits across the first 3 years of their child's life. Neuroimaging will be conducted at 2 weeks, 15 and 24 months (with an accompanying lab visit at 15 months). We will focus on developing white matter tracts that support cognitive processes of emerging executive functions: anterior cingulum (error monitoring); uncinate (joint attention); arcuate fasciculus (language processing) and individual differences in functional brain development, including resting state networks of salience, attention, executive control, and default-mode. At 6 and 24 months of age, an intensive home visit will include observational and objective measures of caregiver behavior, language exposure (via speech recorders) and sleep hygiene (via actigraphy for 7 days). Child cognitive development will be assessed at each assessment and an executive functioning battery will be administered at 36 months of age. This study will be the first to investigate the influence of poverty on emerging executive functioning at age 3 via effects on child neurological development over the first two years of life. In addition, findings will contribute critical information regarding whether specific measures of proximal experience (language exposure, caregiver behavior, child sleep hygiene) may mediate this risk.

PUBLIC HEALTH RELEVANCE: Federal and State governments invest billions of dollars annually in programs that are intended to help “level the playing field” between children who grow up in poverty relative to their peers who do not. However, these investments typically target the 1-2 years prior to kindergarten, which may be too late according to accumulating evidence that (a) early life experience spanning the prenatal period through the first few years of life has a major and lasting impact on children's cognitive development, academic outcomes and eventual opportunities for success and (b) brain structure and major neural networks that support these abilities resemble adult forms by 2 years of age. The proposed study has the potential to inform early intervention and prevention efforts, which is critically important given the severity of the income achievement gap and the limited public health

resources available to address this issue; investments targeting children's early experiences can be more cost effective and impactful for promoting children's learning ability and their eventual opportunity for success.

CRITIQUE 1

Significance: 1
Investigator(s): 1
Innovation: 1
Approach: 1
Environment: 1

Overall Impact: This revised application proposes to examine the effect of poverty on brain development related to executive function in early life and mediating roles of early experiences (sleep, parenting, and language exposure) in the poverty/executive function associations. This prospective longitudinal study will involve 230 women and their children who will complete five assessments across the first three years of the child's life. Poverty has been related to poor neurocognitive development and later academic problems. The scientific premise of this study is strong as it is based on the developmental origins of health and disease model and uses three well-established environmental factors shown to be related to poverty in early life, including parenting, sleep, and language exposure. Findings from this study will generate knowledge to enhance our understanding of early neurological development as an explanatory mechanism for the association between poverty and executive function. The study's direct test of brain development using structural and functional imaging is innovative. The investigator team is excellent covering expertise in parenting, child executive functioning, neuroimaging, and advanced statistical analyses. In the revision, scientific rigor has been strengthened by improving the recruitment approach to ensure a sample representing sufficient variation in poverty. Scientific rigor of this study is strong with comprehensive measures of the three aspects of early experience using multi-method approaches (e.g., questionnaires, behavioral tasks observation, Actigraphy, speech recording), well-thought-out longitudinal analytic plans including multivariate mediational growth curve modeling, and comprehensive power analyses. In addition, the revision offers preliminary findings that provide supporting evidence for all three aims. The potential overall impact is high because this application represents an exciting and innovative study to evaluate the effects of prenatal and postnatal poverty on the development of the brain and executive functioning during the first three years.

1. Significance:

Strengths

- Poverty has been related to poor neurocognitive development and academic problems. This study targets brain development which may be influenced by poverty and early experiences (sleep, parenting, and language exposure) and contribute to executive functioning development early in life (zero to three years). The findings would enhance our understanding of early neurological development as an explanatory mechanism for the association between poverty and executive function.
- The developmental origins of health and disease model guides the hypotheses as an overarching theoretical model, strengthening the scientific premise of the study.
- The mediating roles of the three experiences (sleep, parenting, and language exposure) are supported by the literature and preliminary data.

Weaknesses

- None noted.

2. Investigator(s):

Strengths

- Multiple Principal Investigator Propper has a strong background in child executive functioning, and Multiple Principal Investigator Cox is a leading scientist in developmental psychology and has made invaluable contributions to the parenting and child development research field.
- Other co-investigators cover expertise in neuroimaging and statistical analyses.

Weaknesses

- None noted.

3. Innovation:

Strengths

- This study will be the first to investigate the influence of poverty on executive functioning at age three via effects on neurological development over the first two years of life.
- Direct test of brain development during early ages using structural and functional imaging is innovative.

Weaknesses

- The associations among main study constructs (including poverty, brain, and EF) are well established in the literature.

4. Approach:

Strengths

- The use of Actiwatch and a speech recorder aids objective, comprehensive assessments of sleep patterns and language exposure, respectively.
- Both structural and functional neuroimaging assessments will be performed. For connectivity analyses, both seed-based and independent component analysis approaches are used.
- Power analyses are comprehensive.
- Preliminary findings provide supporting evidence for all three aims.
- Systematic recruitment strategies are presented to ensure that the recruited sample will represent enough families of poverty.

Weaknesses

- In the proposed growth curve model, according to the time sequence, early experiences (caregiving, sleep, and language) measured at 6 months and 24 months predict brain growth rates measured at neonatal/2 weeks, 15 months, and 24 months. Predicting the growth trajectories spanning from neonatal/2 weeks to 24 months by variables measured at 6 and 24 months does not make sense.
- Even in early age, sex differences in brain development may be present, thus need to be considered.

5. Environment:

Strengths

- The Center for Developmental Science, The Neuro Image Research and Analysis Laboratory (NIRAL) at University of North Carolina at Chapel Hill provide excellent settings for the proposed study.

Weaknesses

- None noted.

Protections for Human Subjects:

- No concerns.

Data and Safety Monitoring Plan (Applicable for Clinical Trials Only):

Inclusion of Women, Minorities and Children:

- Sex/Gender: Distribution justified scientifically
- Race/Ethnicity: Distribution justified scientifically
- For NIH-Defined Phase III trials, Plans for valid design and analysis:
- Inclusion/Exclusion of Children under 18: Including ages <18; justified scientifically
- 0-3 year olds, boys and girls, and their mothers; 65 percent White; 35 percent others.

Vertebrate Animals:

Not Applicable (No Vertebrate Animals)

Biohazards:

Not Applicable (No Biohazards)

Resubmission:

- Strengths remain and weaknesses raised in the previous reviews were well addressed.

Budget and Period of Support:

Recommend as Requested:

CRITIQUE 2

Significance: 2
Investigator(s): 1
Innovation: 1
Approach: 2
Environment: 1

Overall Impact: The premise of the study is to examine the influence of early life poverty on young children's executive functioning via effects on brain development in the first two years of life. Language exposure, caregiver behavior, and child sleep hygiene will be examined as mediators of the poverty—brain development association. The methods are rigorous with multiple assessments of the same construct, using observational assessments and established tasks. Mothers will be seen during the

28th week of pregnancy and then infants and primary caregivers will be seen at 5 visits across the first 3 years of the child's life. Neuroimaging will be conducted at 2 weeks, 15 months, and 24 months, with a focus on developing white matter tracts involved with language processing, error monitoring, and joint attention. Functional brain development will also be measured, including resting state networks of default-mode, salience, attention, and executive control. Home visits at 6 and 24 months of age will include observational and objective measures of caregiver behavior, language exposure, and quality of sleep. An executive functioning battery will be administered at 36 months of age. The study has many strengths including the importance of the question, the strong team with complementary areas of expertise, the recruitment during pregnancy, the longitudinal design with gold standard assessments, and the planned missingness design to reduce participant burden. The likely impact of the work is high.

1. Significance:

Strengths

- The scientific premise is to identify brain mediators (reduced cortical volumes and surface area) through which poverty leads to poor executive functioning in 3 year olds.
- The longitudinal design will provide 3 time points of brain scans, allowing the modeling of trajectories of infant brain development.
- By considering the proximal experience that mediate the effects of early poverty on brain development, we may identify actionable targets for subsequent preventative and early intervention efforts.
- Moreover, the most prolific changes in brain development that inform early EF take place from birth to 2 years.

Weaknesses

- None noted

2. Investigator(s):

Strengths

- The team is strong with appropriate expertise. Multiple Principal Investigator Propper has a strong background in studying child poverty and emotion regulation, currently a Research Scientist, Center for Developmental Science, University of North Carolina at Chapel Hill. She is currently principal investigator on two federal grants (R21 from NICHD and a K01 from NIDA).
- Multiple Principal Investigator Short, Assistant Professor at the University of North Carolina in Chapel Hill. Dr. Short's K01 focused on white matter and working memory development in children.
- Other co-investigators cover expertise in neuroimaging and statistical analyses.

Weaknesses

- None noted

3. Innovation:

Strengths

- The focus on how early life experiences and cognitive and neural development contribute to young children's executive functioning is unique and addresses a gap in knowledge about brain development from birth to age 2 years.

Weaknesses

- None noted

4. Approach:

Strengths

- The approach is vigorous in that recruitment occurs before the birth of the child (230 women are recruited from the hospital around 28 weeks of pregnancy), and objective measures are utilized.
- The planned missing design is efficient and reduces participant burden (families participate in 2 of 3 imaging visits).
- Objective measures of sleep (actiwatch), language exposure (LENA), and parenting are considered as mediators of the poverty—brain development link.
- Structural and functional neuroimaging including diffusion tensor imaging and resting state fMRI is used at multiple time points across infancy and early childhood (2 weeks, 15 months and 24 months).
- A computerized battery of EF tasks (EF Touch) is used to measure inhibitory control, attentional shifting and working memory.
- Impressive results of preliminary studies

Weaknesses

- None noted

5. Environment:

Strengths

- The Center for Developmental Science, The Neuro Image Research and Analysis Laboratory (NIRAL) at University of North Carolina at Chapel Hill are excellent environments for the proposed study.

Weaknesses

- None noted

Protections for Human Subjects:

Acceptable Risks and/or Adequate Protections

- Adequate protections are in place. Ear protection is used during the MRI scans, participants are swaddled and scanned during sleep, and parents are allowed to stay with their child.

Data and Safety Monitoring Plan (Applicable for Clinical Trials Only):

Not Applicable (No Clinical Trials)

Inclusion of Women, Minorities and Children:

- Sex/Gender: Distribution justified scientifically
- Race/Ethnicity: Distribution justified scientifically
- For NIH-Defined Phase III trials, Plans for valid design and analysis: Not applicable
- Inclusion/Exclusion of Children under 18: Including ages <18; justified scientifically
- 0-3 year olds, boys and girls, and their mothers; 65 percent White; 26 percent African American.

Vertebrate Animals:

Not Applicable (No Vertebrate Animals)

Biohazards:

Not Applicable (No Biohazards)

Resubmission:

- Responsive to the original reviews

Resource Sharing Plans:

Acceptable

- De-identified data will be shared through NIH's DNAR database in the last quarter of year 5 of the grant

Budget and Period of Support

Recommend as Requested

CRITIQUE 3

Significance: 2
Investigator(s): 3
Innovation: 1
Approach: 3
Environment: 1

Overall Impact: The proposed study aims to examine the effects of poverty on executive function (EF) from birth to preschool age with an examination of brain structure and function, as well as child sleep, parenting behaviors, and word exposure as mediators of pathways from poverty to EF. The work is highly significant as children from low SES background start preschool behind in academic and socio-emotional skills, many of which are promoted by EF. Deficits in EF in preschool predict health, wealth, and violence into adulthood, making the issue highly significant. Though there is much desire to “level the playing field” for children early, little is known about how poverty may be affecting early brain development during both the pre- and postnatal period. Thus, the results of this work can inform policy and early prevention efforts. The proposed project is quite innovative in using MRI with very young children and combining this approach with a developmental science approach to measuring poverty, environmental mediators, and EF outcomes longitudinally. The investigative team has complimentary areas of expertise across the wide range of areas covered by this application. In particular, both principal investigators have very separate and complimentary experience and there is a good balance of early career and senior investigators. Moreover, the neuroimaging half of the team have been pioneers in the very difficult task of using MRI (and fMRI in particular) with babies. The application is responsive to previous reviews and the study is improved through a sampling plan likely to yield a locally representative sample that contains ethnic and socioeconomic diversity. Other strengths of the study include an innovative planned missingness design, the use of longitudinal imaging and constructing growth curves using MRI data, the ability to separate prenatal (and genetic) experiential effects on the brain from early-life contextual effects through the use of multiple imaging timepoints and modeling change over time, and cutting edge measurement of mediators (using observational

measures of parenting, actigraphy for sleep, and LENA for word exposure). The overall innovation and significance of the proposed project is slightly diminished by some minor weaknesses and details that are under-described in the application including a very large Investigative team with seemingly overlapping areas of expertise (which may make coordination of the team difficult), lack of details about how high quality infant imaging data is achieved (i.e., the team clearly is expert at this task, but there is little detail about how the infants are kept still and happy – are they asleep? If so, how is this achieved and is it really a “resting state” scan?), and a lack of details about how the neuroimaging data will be reduced/focused on and put into SEMs (extracted from only a few regions of interest? Using what methods?). These weaknesses are relatively minor. The study has a strong scientific premise and is guided well by the literature reviewed. The study also appears to be adequately powered and potentially well-sampled, which assures high rigor and potential reproducibility. Sex as a biological variable is not featured prominently, but is addressed adequately by recruitment of boys and girls. Finally, the institutions support this research are strong with adequate resources.

Protections for Human Subjects:

Acceptable Risks and/or Adequate Protections

- The protection of subjects plan is thoughtful and careful in protecting family information

Data and Safety Monitoring Plan (Applicable for Clinical Trials Only):

Not Applicable (No Clinical Trials)

Inclusion of Women, Minorities and Children:

- Sex/Gender: Distribution justified scientifically
- Race/Ethnicity: Distribution justified scientifically
- For NIH-Defined Phase III trials, Plans for valid design and analysis: Not applicable
- Inclusion/Exclusion of Children under 18: Including ages <18; justified scientifically
- The sample will be diverse ethnically and socioeconomically and contain half boys and half girls as well as parents, which are all justified scientifically.

Vertebrate Animals:

Not Applicable (No Vertebrate Animals)

Biohazards:

Not Applicable (No Biohazards)

Resubmission:

- The application was responsive to previous reviews and the proposed study is now improved, making the research stronger with higher rigor.

Budget and Period of Support:

Recommend as Requested

THE FOLLOWING SECTIONS WERE PREPARED BY THE SCIENTIFIC REVIEW OFFICER TO SUMMARIZE THE OUTCOME OF DISCUSSIONS OF THE REVIEW COMMITTEE, OR REVIEWERS' WRITTEN CRITIQUES, ON THE FOLLOWING ISSUES:

PROTECTION OF HUMAN SUBJECTS: ACCEPTABLE

INCLUSION OF WOMEN PLAN: ACCEPTABLE

INCLUSION OF MINORITIES PLAN: ACCEPTABLE

INCLUSION OF CHILDREN PLAN: ACCEPTABLE

COMMITTEE BUDGET RECOMMENDATIONS: The budget was recommended as requested.

Footnotes for 1 R01 HD091148-01A1; PI Name: Propper, Cathi Barbra

NIH has modified its policy regarding the receipt of resubmissions (amended applications). See Guide Notice NOT-OD-14-074 at http://grants.nih.gov/grants/guide/notice-files/NOT-OD-14-074.html. The impact/priority score is calculated after discussion of an application by averaging the overall scores (1-9) given by all voting reviewers on the committee and multiplying by 10. The criterion scores are submitted prior to the meeting by the individual reviewers assigned to an application, and are not discussed specifically at the review meeting or calculated into the overall impact score. Some applications also receive a percentile ranking. For details on the review process, see http://grants.nih.gov/grants/peer_review_process.htm#scoring.

MEETING ROSTER

Psychosocial Development, Risk and Prevention Study Section
Risk, Prevention and Health Behavior Integrated Review Group
CENTER FOR SCIENTIFIC REVIEW
PDRP
06/08/2017 - 06/09/2017

Notice of NIH Policy to All Applicants: Meeting rosters are provided for information purposes only. Applicant investigators and institutional officials must not communicate directly with study section members about an application before or after the review. Failure to observe this policy will create a serious breach of integrity in the peer review process, and may lead to actions outlined in NOT-OD-14-073 at https://grants.nih.gov/grants/guide/notice-files/NOT-OD-14-073.html and NOT-OD-15-106 at https://grants.nih.gov/grants/guide/notice-files/NOT-OD-15-106.html, including removal of the application from immediate review.

CHAIRPERSON(S)

LANSFORD, JENNIFER E, PHD
RESEARCH PROFESSOR
CENTER FOR CHILD AND FAMILY POLICY
DUKE UNIVERSITY
DURHAM, NC 27708

MEMBERS

BRIGGS-GOWAN, MARGARET J, PHD
ASSOCIATE PROFESSOR
DEPARTMENT OF PSYCHIATRY
UNIVERSITY OF CONNECTICUT HEALTH CENTER
FARMINGTON, CT 06030

BROPHY-HERB, HOLLY E, PHD
PROFESSOR
DEPARTMENT OF HUMAN DEVELOPMENT
AND FAMILY STUDIES
MICHIGAN STATE UNIVERSITY
EAST LANSING, MI 48824

BUSS, KRISTIN A, PHD *
PROFESSOR
DEPARTMENT OF PSYCHOLOGY
THE PENNSYLVANIA STATE UNIVERSITY
UNIVERSITY PARK, PA 16802

CRANGLE, COLLEEN E, PHD *
ASSOCIATE PROFESSOR/ ASSOCIATE EDITOR
PUBLIC LIBRARY OF SCIENCE
INFORMATION SCIENCES
UNIVERSITY OF LOUISVILLE
SCHOOL OF PUBLIC HEALTH
SAN FRANCISCO, CA 94111

DOZIER, MARY, PHD *
PROFESSOR
DEPARTMENT OF PSYCHOLOGICAL AND BRAIN SCIENCES
UNIVERSITY OF DELAWARE
NEWARK, DE 19716

EIDEN, RINA D, PHD
SENIOR RESEARCH SCIENTIST
RESEARCH INSTITUTE ON ADDICTIONS
STATE UNIVERSITY OF NEW YORK AT BUFFALO
BUFFALO, NY 14203

FOWLER, PATRICK J, PHD *
ASSOCIATE PROFESSOR
THE BROWN SCHOOL
WASHINGTON UNIVERSITY IN ST. LOUIS
ST. LOUIS, MO 63130

GRABER, JULIA A, PHD *
PROFESSOR
DEPARTMENT OF PSYCHOLOGY
UNIVERSITY OF FLORIDA
GAINESVILLE, FL 32611

GRAHAM, SANDRA H, PHD *
PROFESSOR
DEPARTMENT OF EDUCATION
UNIVERSITY OF CALIFORNIA, LOS ANGELES
LOS ANGELES, CA 90095

HYDE, LUKE W, PHD *
ASSISTANT PROFESSOR
DEPARTMENT OF PSYCHOLOGY
CENTER FOR HUMAN GROWTH AND DEVELOPMENT
UNIVERSITY OF MICHIGAN
ANN ARBOR, MI 48104

JAFFEE, SARA R, PHD *
PROFESSOR
DEPARTMENT OF PSYCOLOGY
UNIVERSITY OF PENNSYLVANIA
PHILADELPHIA, PA 19104

JOE, SEAN, PHD *
ASSOCIATE DEAN FOR FACULTY AND RESEARCH
SCHOOL OF SOCIAL WORK
WASHINGTON UNIVERSITY
ST. LOUIS, MO 63130

KIM-SPOON, JUNGMEEN, PHD
PROFESSOR
DEPARTMENT OF PSYCHOLOGY
VIRGINIA TECH
BLACKSBURG, VA 24061

LEERKES, ESTHER M, PHD
PROFESSOR
DEPARTMENT OF HUMAN DEVELOPMENT
AND FAMILY STUDIES
UNIVERSITY OF NORTH CAROLINA AT GREENSBORO
GREENSBORO, NC 27402-6170

LEJUEZ, CARL W, PHD
DEAN
COLLEGE OF LIBERAL ARTS AND SCIENCES
UNIVERSITY OF KANSAS
LAWERENCE, KS 66045

LEMERY-CHALFANT, KATHRYN, PHD
PROFESSOR
DEPARTMENT OF PSYCHOLOGY
ARIZONA STATE UNIVERSITY
TEMPE, AZ 85287

LIGHTFOOT, MARGUERITA A, PHD
PROFESSOR
DEPARTMENT OF MEDICINE
SCHOOL OF MEDICINE
UNIVERSITY OF CALIFORNIA, SAN FRANCISCO
SAN FRANCISCO, CA 94105

MCCLOSKEY, MICHAEL S, PHD *
ASSOCIATE PROFESSOR
DEPARTMENT OF PSYCHOLOGY
TEMPLE UNIVERSITY
PHILDELPHIA, PA 19122

MILLER, ALISON L, PHD
ASSOCIATE PROFESSOR
DEPARTMENT OF HEALTH BEHAVIOR
AND HEALTH EDUCATION
SCHOOL OF PUBLIC HEALTH
UNIVERSITY OF MICHIGAN
ANN ARBOR, MI 48109

MURRY, VELMA M, PHD
JOE B WYATT DISTINGUISHED RESEARCH PROFESSOR
DEPARTMENT OF HUMAN AND ORGANIZATIONAL
DEVELOPMENT
VANDERBILT UNIVERSITY
NASHVILLE, TN 37203

OBASI, EZEMENARI M, PHD
ASSOCIATE PROFESSOR
DEPARTMENT OF PSYCHOLOGICAL HEALTH
AND LEARNING SCIENCES
COLLEGE OF EDUCATION
UNIVERSITY OF HOUSTON
HOUSTON, TX 77204

ROCHE, KATHLEEN M, PHD *
ASSOCIATE PROFESSOR
DEPARTMENT OF PREVENTION AND COMMUNITY HEALTH
MILKEN INSTITUTE SCHOOL OF PUBLIC HEALTH
GEORGE WASHINGTON UNIVERSITY
WASHINGTON, DC 20052

ROISMAN, GLENN I, PHD
PROFESSOR
INSTITUTE OF CHILD DEVELOPMENT
UNIVERSITY OF MINNESOTA
MINNEAPOLIS, MN 55455

ROTHMAN, EMILY F, SCD *
ASSOCIATE PROFESSOR
SCHOOL OF PUBLIC HEALTH
BOSTON UNIVERSITY
BOSTON, MA 02118

SANTA MARIA, DIANE M, BSN, DRPH, RN *
ASSISTANT PROFESSOR
SCHOOL OF NURSING
UNIVERSITY OF TEXAS HEALTH SCIENCE CENTER
HOUSTON, TX 77030

SHAW, DANIEL S, PHD
DISTINGUISHED PROFESSOR
DEPARTMENT OF PSYCHOLOGY
UNIVERSITY OF PITTSBURGH
PITTSBURGH, PA 15260

SHORTT, JOANN WU, PHD
SENIOR RESEARCH SCIENTIST
OREGON SOCIAL LEARNING CENTER
EUGENE , OR 97401

SKOWRON, ELIZABETH A, PHD
PROFESSOR
DEPARTMENT OF COUNSELING PSYCHOLOGY
AND HUMAN SERVICES
PREVENTION SCIENCE INSTITUTE
UNIVERSITY OF OREGON
EUGENE, OR 97403

SPIRITO, ANTHONY, PHD
PROFESSOR VICE CHAIR
DEPARTMENT OF PSYCHIATRY
AND HUMAN BEHAVIOR
BROWN UNIVERSITY
PROVIDENCE , RI 02912

SUCHMAN, NANCY E, PHD
ASSOCIATE PROFESSOR
DEPARTMENT OF PSYCHIATRY
AND YALE CHILD STUDY CENTER
YALE UNIVERSITY SCHOOL OF MEDICINE
NEW HAVEN, CT 06511

TELZER, EVA H, PHD
ASSISTANT PROFESSOR
DEPARTMENT OF PSYCHOLOGY AND NEUROSCIENCE
UNIVERSITY OF NORTH CAROLINA, CHAPEL HILL
CHAPEL HILL, NC 27599

VALOIS, ROBERT F, PHD *
PROFESSOR
DEPARTMENT OF HEALTH PROMOTION
EDUCATION AND BEHAVIOR
ARNOLD SCHOOL OF PUBLIC HEALTH
UNIVERSITY OF SOUTH CAROLINA
COLUMBIA, SC 29208

WU, ELWIN, PHD *
ASSOCIATE PROFESSOR
SCHOOL OF SOCIAL WORK
COLUMBIA UNIVERSITY
NEW YORK, NY 10027

YOUNG, JAMI F, PHD
ASSOCIATE PROFESSOR
CHILDRENS HOSPITAL
AT PHILADELPHIA
PHILADELPHIA, PA 19146

MAIL REVIEWER(S)

GHOSH, DEBASHIS, PHD
PROFESSOR AND CHAIR
DEPARTMENT OF BIOSTATISTICS AND INFORMATICS
COLORADO SCHOOL OF PUBLIC HEALTH
UNIVERSITY OF COLORADO ANSCHUTZ MEDICAL CAMPUS
AURORA, CO 80045

SCIENTIFIC REVIEW OFFICER

RILEY, ANNA L, PHD
SCIENTIFIC REVIEW OFFICER
CENTER FOR SCIENTIFIC REVIEW
NATIONAL INSTITUTES OF HEALTH
BETHESDA, MD 20892

EXTRAMURAL SUPPORT ASSISTANT

WATTS, MELISSA D
EXTRAMURAL SUPPORT ASSISTANT
CENTER FOR SCIENTIFIC REVIEW
NATIONAL INSTITUTE FOR HEALTH
BETHESDA, MD 20892

* Temporary Member. For grant applications, temporary members may participate in the entire meeting or may review only selected applications as needed.

Consultants are required to absent themselves from the room during the review of any application if their presence would constitute or appear to constitute a conflict of interest.
